# Comparative characterization of mesenchymal stem cells from eGFP transgenic and non-transgenic mice

**DOI:** 10.1186/1471-2121-10-3

**Published:** 2009-01-13

**Authors:** Cynthia B Ripoll, Bruce A Bunnell

**Affiliations:** 1 Department of Pharmacology, Tulane University Health Sciences Center, New Orleans, LA, USA; 2 Division of Gene Therapy, Tulane National Primate Research Center, Covington, LA, USA; 3 Center for Gene Therapy, Tulane University Health Sciences Center, New Orleans, LA, USA

## Abstract

**Background:**

Adipose derived- and bone marrow-derived murine mesenchymal stem cells (mMSCs) may be used to study stem cell properties in an *in vivo *setting for the purposes of evaluating therapeutic strategies that may have clinical applications in the future. If these cells are to be used for transplantation, the question arises of how to track the administered cells. One solution to this problem is to transplant cells with an easily identifiable genetic marker such as enhanced green fluorescent protein (eGFP). This protein is fluorescent and therefore does not require a chemical substrate for identification and can be visualized in living cells. This study seeks to characterize and compare adipose derived- and bone marrow-derived stem cells from C57Bl/6 mice and eGFP transgenic C57Bl/6 mice.

**Results:**

The expression of eGFP does not appear to affect the ability to differentiate along adipogenic or osteogenic lineages; however it appears that the tissue of origin can influence differentiation capabilities. The presence of eGFP had no effect on cell surface marker expression, and mMSCs derived from both bone marrow and adipose tissue had similar surface marker profiles. There were no significant differences between transgenic and non-transgenic mMSCs.

**Conclusion:**

Murine adipose derived and bone marrow derived mesenchymal stem cells from non-transgenic and eGFP transgenic C57Bl/6 mice have very similar characterization profiles. The availability of mesenchymal stem cells stably expressing a genetic reporter has important applications for the advancement of stem cell research.

## Background

Mesenchymal stem cells can be broadly defined as a population of cells that have the ability to self-renew, adhere to plastic, and to differentiate into one or more specialized cell types [1-3]. Tissue specific stem cells, such as mesenchymal stem cells (MSCs) derived from bone marrow or adipose tissue, were initially thought to have a differentiation capacity limited to the tissue of origin. However, recent studies have shown that these cells may have the ability to differentiate into cells of mesodermal, endodermal, and ectodermal origins [4-8]. The term "mesenchymal stem cell" most often refers to stem cells derived from the stromal fraction of bone marrow, but can also be applied to stem cells derived from adipose tissue. Adipose tissue is also derived from the mesenchyme and contains a supportive stroma that is easily isolated [4,9]. Both adipose derived stem cells (ASCs) and bone marrow derived stem cells (BMSCs) can be easily harvested in significant numbers and exhibit stable growth and proliferation in culture.

MSCs have garnered increasing amounts of attention for their potential use in cell-based therapies and in regenerative medicine because of several attractive features: (1) they are easily isolated by bone marrow aspiration or liposuction under local anesthesia; (2) they can be extensively expanded in culture without loss of differentiation potential; (3) MSCs can differentiate into multiple cell types; (4) MSCs have the ability to seek out sites of tissue injury and repair the tissue by differentiating to replace injured cells or by creating an environment favorable for the repair of damaged tissue by endogenous cells [10]. The utilization of MSCs derived from the adipose tissue or bone marrow of mice is appealing for use in extensive studies in the field of adult stem cell research due to the low cost of maintaining mice. These murine MSCs (mMSCs) may be used to study stem cell properties in an *in vivo *setting for the purposes of evaluating therapeutic strategies that may have clinical applications in the future.

Whenever cells are to be used in a transplantation scenario, the question of how to track the engraftment, persistence, and differentiation of the administered cells arises. Tracking the cells should help determine whether a tissue is repaired by the transplanted stem cells, by activation of host stem cells, or by host cells recruited by the transplanted stem cells to the site of injury.

Several reporter gene or tracking strategies are currently being developed to assist investigators with experimental stem cell therapies. Cells can be transfected or transduced with vectors carrying a fluorescent transgene, but these methods are limited by the efficiency of gene transfer and retention [11-13]. Other reporter genes may encode enzymes such as β-galactosidase (lacZ), chloramphenicol acetyltransferase, and firefly luciferase. These enzymes require the addition of substrates to visualize labeled cells, which may lead to background staining if endogenous enzymes can also process the added substrate [14-16]. Fluorescent DNA binding dyes such as bis-benzimide are also available [17], but are also limited by binding efficiencies and background fluorescence from DNA released from dead cells. Transplanting cells harvested from a male donor into a female host and searching for the Y-chromosome is also a simple and effective strategy for tracking cells, but this technique cannot be utilized in living cells.

One solution to this problem is to transplant cells with an easily detectable or identifiable genetic reporter element. An ideal reporter gene would (1) cause no adverse effects to the stem cells or host development; (2) be ubiquitously expressed in all transplanted cells; (3) be precisely localized to transplanted cells; (4) be reliably detected in living cells, tissue mounts, and fixed and paraffin-embedded sections; and (5) be compatible with visualization of other markers [15]. For these purposes, an ideal approach is to harvest stem cells from a transgenic mouse strain expressing a transgene marker. These cells can be transplanted into non-transgenic mice without the complication of background staining.

One such transgene marker is enhanced green fluorescent protein (eGFP). eGFP is an excellent reporter because it can be visualized in living cells in culture and has already been useful in monitoring gene expression, protein-protein interactions, and trafficking and localization *in vivo *[15,18-21]. eGFP-expressing transgenic mice are readily available and are a possible source for murine ASCs (mASCs) and murine BMSCs (mBMSCs) which constitutively express eGFP [21]. These mice are uniformly green with the exceptions of hair and red blood cells [12].

The efficiency of establishing stable cell lines with an integrated eGFP gene is normally low, therefore cells from eGFP transgenic mice may prove more useful and easier to culture [22]. However, employing eGFP as a means of tracking cells has a couple drawbacks. One limitation is that eGFP/GFP is thought to induce cell death [12]. Intense excitation of the protein *in vitro *for extended periods of time can generate toxic free radicals, and the presence of GFP may also lead to increased DNA methylation [22,23]. eGFP was also designed to be expressed in the cytosol of a cell, which could lead to toxic effects if present in high concentrations. In spite of these potential obstacles, cell lines from transgenic mice often look normal and healthy even though they express significant amounts of eGFP [14].

This study seeks to characterize and compare four different groups of mMSCs consisting of mASCs and mBMSCs derived from C57Bl/6 mice and mASCs and mBMSCs derived from eGFP transgenic C57Bl/6 mice (GFPTgASCs and GFPTgBMSCs). Characteristics such as differentiation potential, colony forming unit capabilities, cell surface marker profiles, and growth kinetics of these four groups of cells were evaluated in order to give future investigators a clearer understanding of the properties of eGFP transgenic mMSCs (GFPTgMSCs). Mesenchymal stem cells derived from eGFP transgenic mice may prove useful in future testing and development of stem cell therapies and regenerative medicine applications because of their inherent stem cell properties and constitutive expression of the fluorescent protein eGFP.

## Results

### Morphology of Transgenic mMSCs

**Figure 1:**
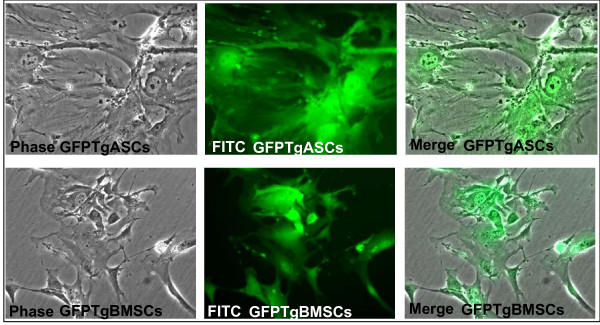
**Morphology and fluorescence of GFPTgASCs and GFPTgBMSCs**. Top: ASCs derived from eGFP+ transgenic C57Bl/6 mice (P4), Bottom: BMSCs derived from eGFP+ transgenic C57Bl/6 mice (P3). 20× magnification.

The morphology of transgenic mMSCs is consistent with that of normal mMSCs in that they are fibroblastic in appearance. mBMSCs and GFPTgBMSCs seem to be more spindle-shaped than mASCs or GFPTgASCs, thus individual mASCs and GFPTgASCs require more surface area on the culture dish due to their larger and more flattened shape. GFPTgMSCs fluoresce when examined through a FITC filter (Figure 1), and remained fluorescent and maintained morphology through passage 10 (data not shown). The mMSCs were not cultured past passage 10 for this study.

### Differentiation Assays

**Figure 2:**
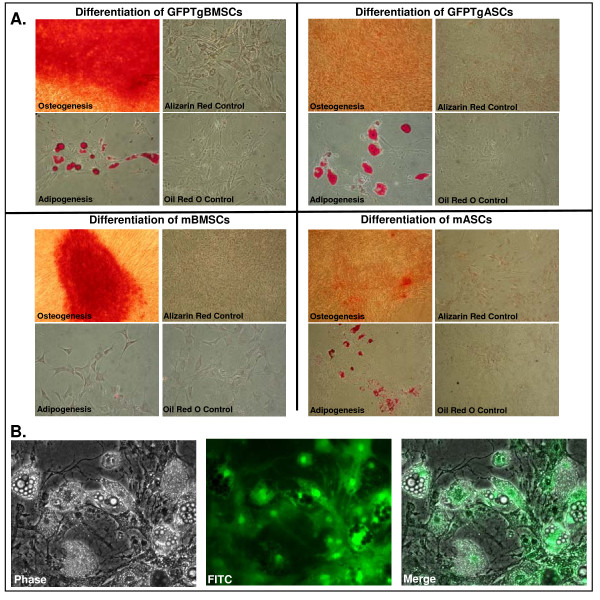
**A. Differentiation of mMSCs and GFPTgMSCs along mesodermal lineages**. Cells were incubated in CEM and then transferred to adipogenic or osteogenic media for 14 days. All cells were passage 9 or lower. Cells which formed lipid vacuoles were stained with Oil Red-O while mineralization in osteogenic-differentiated cells was revealed with Alizarin Red staining.B. GFPTgMSCs retain fluorescence after differentiation. MSCs derived from eGFP+ transgenic C57Bl/6 mice (P6) retain fluorescence even after stimulated to undergo adipogenic differentiation. 20× magnification.

**Figure 3:**
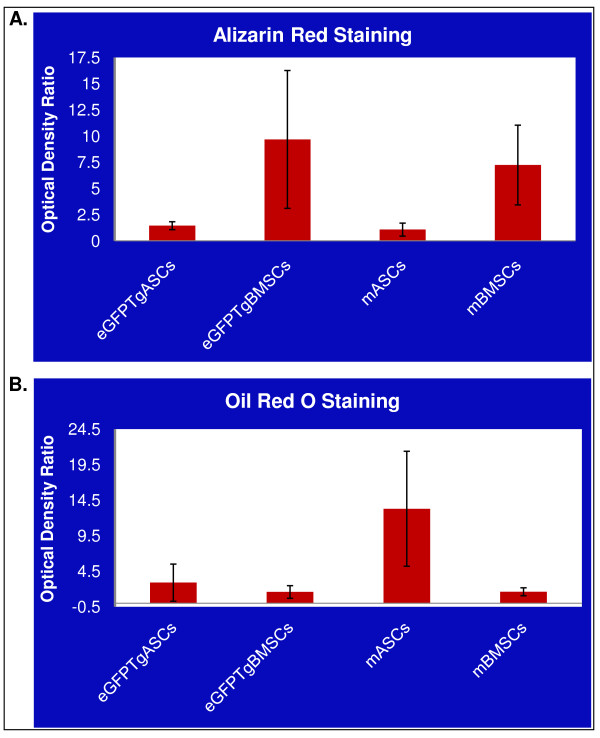
**Quantitative differentiation of mMSCs and GFPTgMSCs along osteogenic and adipogenic lineages**. A. Cells were incubated in CEM and then transferred to osteogenic media for 14 days. The cells were then stained with Alizarin Red and de-stained with 10% cetylpyridinium chloride. Optical density (OD) was measured at 560 nm and normalized to protein content. Graphs represent the ratio of normalized OD of differentiated cells and normalized OD of control cells. B. Cells were incubated in CEM and then transferred to adipogenic media for 14 days. The cells were then stained with Oil Red O and de-stained with isopropanol. OD was measured at 520 nm and normalized to protein content. Graphs represent the ratio of normalized OD of differentiated cells and normalized OD of control cells.

When cultured mMSCs (mBMSCs, mASCs, GFPTgASCs, and GFPTgBMSCs) were exposed to an osteogenic induction medium, they aggregated and formed calcium deposits after 2 weeks. An alizarin red stain for precipitated calcium salt was performed on differentiated cells. The mMSCs (Passage 9 or lower) readily underwent osteogenic differentiation into mineralizing cells, and the transgenic mMSCs and normal mMSCs differentiated in a similar manner (Figure 2A). However, quantitation of the levels of differentiation in these cell populations indicated that the mBMSCs (Optical Density (OD) ratio = 7.2 +/- 3.8) and GFPTgBMSCs (OD ratio = 9.7 +/- 6.6) appeared to differentiate into mineralizing cells to a greater degree than the mASCs (OD ratio = 1.1 +/- 0.6) and GFPTgASCs (OD ratio = 1.45 +/- 0.4) (p < 0.05) (Figure 3A). Graphs represent the ratio of normalized OD of differentiated cells and normalized OD of control cells.

The ability to differentiate into adipocytes in response to 2 weeks of culture in adipogenic induction media was similar between the mMSCs and transgenic mMSCs (Passage 9 or lower). The mASCs (OD ratio = 13.3 +/- 8.1) and GFPTgASCs (OD ratio = 2.9 +/- 2.6) differentiated along adipogenic lineages and had slightly higher levels of lipid accumulation when stained with Oil Red O than mBMSCs (OD ratio = 1.6 +/- 0.6) or GFPTgBMSCs (OD ratio = 1.6 +/- 0.9), though this difference was not statistically significant (p > 0.05) (Figure 3B). Graphs represent the ratio of normalized OD of differentiated cells and normalized OD of control cells.

GFPTgASCs and GFPTgBMSCs retain their ability to fluoresce when examined through a FITC filter even after undergoing adipogenic (Figure 2B) or osteogenic differentiation assays. The fluorescence after osteogenic differentiation is not pictured because osteogenic differentiation is not easily illustrated without an Alizarin Red stain, which consequently quenches the fluorescence due to the low pH of the stain.

A chondrogenic differentiation assay was performed with all mMSCs (data not shown), and all cell types formed stable pellets. However, the mMSCs produced little proteoglycans in response to chondrogenic differentiation media. The inability of mMSCs from C57Bl/6 and other strains of mice to undergo efficient chondrogenesis has been noted previously [24] and may be attributed to the lack of murine specific cytokines.

### FACS analysis

**Figure 4:**
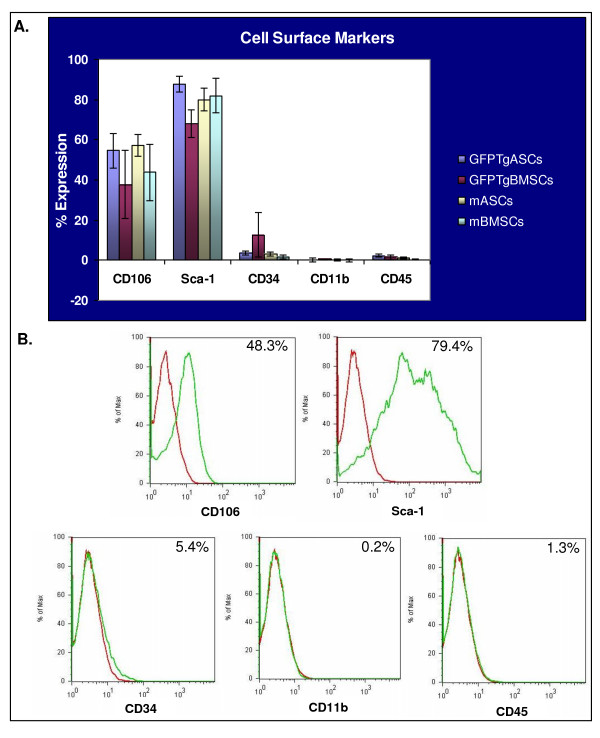
**Immunophenotypic profile of mMSCs and GFPTgMSCs**. A. Stem cells (P10 or lower) were incubated with antibodies for CD106, Sca-1, CD34, CD11b, or CD45 and assayed by FACS. Each antibody was tested individually and with isotype controls. B. Representative plots are shown with percentages corresponding to the average profile of all cell types. Red plot lines: isotype control; green plot: stem cells.

Flow cytometric analysis of cell surface markers revealed that all of the mMSCs and GFPTgMSCs were virtually identical in their patterns of expression (Figure 4A). Cells were tested for expression of markers such as CD106 (VCAM-1), Sca-1 (stem cell antigen-1; Ly6A/E), CD34 (mucosialin), CD11b (Mac-1α; Integrin alpha M), and CD45 (leukocyte common antigen; Ly-5). All cells exhibited very low expression of epitopes generally associated with hematopoietic cells such as CD34 (5.4% +/- 5.0%), CD11b (0.2% +/- 0.3%), and CD45 (1.3% +/- 0.8%) [25]. Approximately half of the cells expressed CD106 (48.3% +/- 9.2%), which is a marker generally associated with mesenchymal stem cells [25]. Most cells expressed Sca-1 (79.4% +/- 8.3%), which is a marker associated with MSCs, but also with hematopoietic and endothelial progenitors [11,25,26] (Figure 4B).

### Growth Kinetics

**Figure 5:**
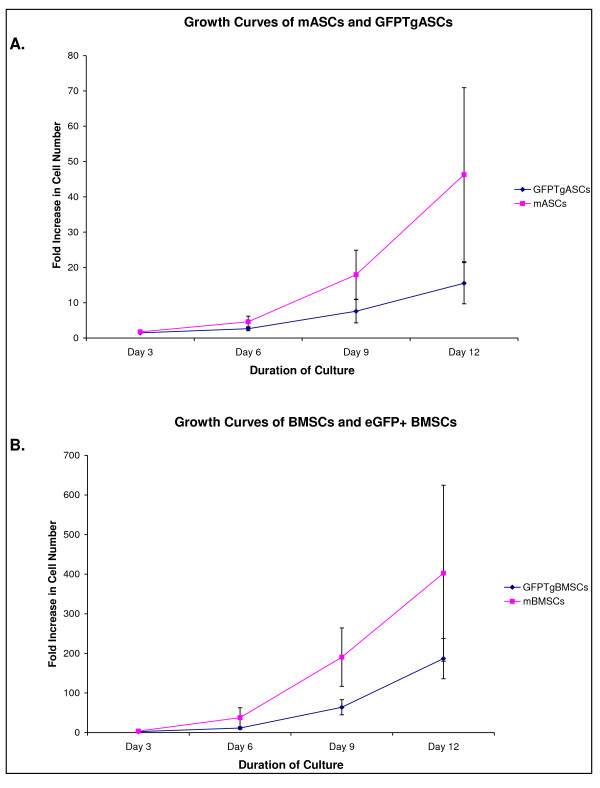
**A. Growth Curves of mASCs and GFPTgASCs**. Cells were plated at an original density of 100 cells/cm^2 ^and cultured for 12 days. The difference in growth rate between mASCs and GFPTgASCs is not statistically significant (p > 0.05). B. Growth Curves of mBMSCs and GFPTgBMSCs. Cells were plated at an original density of 100 cells/cm^2 ^and cultured for 12 days. The difference in growth rate between mBMSCs and GFPTgBMSCs is not statistically significant (p > 0.05).

All mMSCs and GFPTgMSCs were plated at a density of 100 cells/cm^2 ^in 56.7 cm^2 ^plates and cultured for 12 days. Fold increase in density was analyzed every 3 days throughout the culture and calculated by comparing the density at each time point to the original plating density of 100 cells/cm^2^. As illustrated in Figure 5, the four groups of mMSCs varied greatly from one another in their growth rates. mASCs had a 46-fold increase (+/-24.6) in cell number while GFPTgASCs only had a 16-fold increase (+/- 5.8) within the same time period (Figure 5A). Similarly, mBMSCs had a 402-fold increase (+/- 222.3) in cell number while GFPTgBMSCs only had a 187-fold increase (+/- 51.1) (Figure 5B).

### Colony Forming Unit Assay

**Figure 6:**
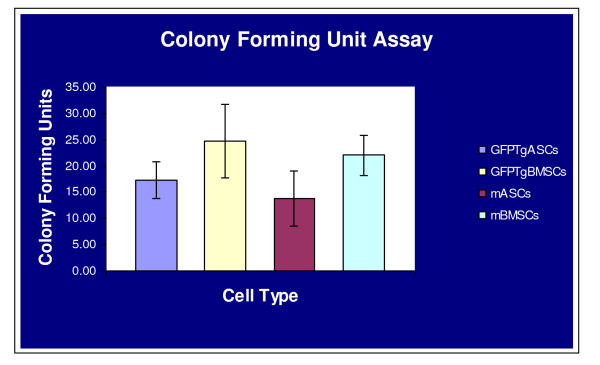
**Colony Forming Unit Assay**. 100 cells were plated in a 56.7 cm^2 ^culture dish and incubated for 14 days. Cells were stained with 3% Crystal Violet, and colonies 2 mm or larger were counted. The ability to form colonies was similar between all cell types (p > 0.05).

Assays for colony forming units offer a convenient means of assessing the proliferative capacity that MSCs retain after the cells have been expanded in culture [27,28]. The proliferative capacity of mMSCs and GFPTgMSCs was examined by comparing CFU assay results between these groups of cells. No statistically significant differences were found between mASCs (13.8 +/- 10.5), GFPTgASCs (17.2 +/- 8.9 CFUs), mBMSCs (22.0 +/- 6.7), or GFPTgBMSCs (24.7 +/- 12.2) in their abilities to generate colony forming units (Figure 6).

## Discussion

Isolation of MSCs from mice is often more difficult than from other species [29]. Only a small amount of bone marrow and adipose tissue is available per mouse and harvesting these tissues can sometimes be problematic. Often there is unwanted growth of non-mesenchymal cells that can take over the culture. However, these challenges can be overcome to develop murine mesenchymal stem cell lines with consistently similar biologic properties. This study focused on comparing adipose derived and bone marrow derived stem cells from eGFP transgenic C57Bl/6 mice and normal C57Bl/6 mice. Even though these cells were isolated from 3–4 donors for each cell type, the characterizations of these four different cell types are relatively comparable with few differences.

mMSCs from eGFP transgenic mice are similar in their fibroblastic morphology and in differentiation capability to those from non-transgenic mice. However, when mASCs and GFPTgASCs are compared with mBMSCs and GFPTgBMSCs there are clear distinctions. mASCs and GFPTgASCs appear more flattened and wider in diameter when compared to mBMSCs and GFPTgBMSCs. Both types of mBMSCs undergo osteogenic differentiation more readily than mASCs or GFPTgASCs. These distinctions point to tissue-specific differences between murine stem cells derived from adipose tissue and bone marrow. Perhaps mASCs contain more adipogenic committed progenitor cells compared with mBMSCs, and mBMSCs contain more osteogenic committed progenitor cells compared with mASCs. Finally, the expression of eGFP does not seem to affect the ability to differentiate along adipogenic or osteogenic lineages, and cells retain their fluorescence even after undergoing differentiation.

All four cell types were also analyzed by flow cytometry for specific cell surface markers. Cells were tested for expression of markers such as CD106, Sca-1, CD34, CD11b, and CD45. All cells exhibited very low expression of epitopes generally associated with hematopoietic cells such as CD34, CD11b, and CD45 [25]. These results suggest that the cultures were virtually free of hematopoietic cells. Approximately half of the cells expressed CD106, which is a marker generally associated with mesenchymal stem cells [25]. Most cells expressed Sca-1, which is a marker associated with MSCs and also with hematopoietic and endothelial progenitors [11,25,26]. The cell surface marker profiles of these transgenic and non-transgenic mASCs and mBMSCs are very similar to those previously reported [9,24,30]. The presence of eGFP appeared to have no effect on surface marker expression. Interestingly, mMSCs derived from both bone marrow and adipose tissue had similar cell surface marker profiles. This is not surprising since both cell types are isolated from the stromal cell fraction of the original tissue and are isolated based on their adherence to plastic [9].

Comparisons of growth curves from the four cell types yielded interesting results. First, the GFPTgMSCs seemed to grow more slowly than their non-transgenic counterparts. However, the differences were not statistically significant due to variation among donors. Perhaps increasing the sample size of donors would decrease the amount of variation and illustrate a significant difference in growth kinetics. Secondly, it seems that adipose-derived murine stem cells grow more slowly than those derived from bone marrow. Again, the differences were not statistically significant due to variation among donors. Perhaps the media used to culture mASCs should be customized to maximize growth potential.

Finally, all four cell types readily formed single-cell derived colonies when plated at low density. The presence of eGFP in the transgenic cells did not seem to affect the proliferative capacity of the cells after expansion in culture.

## Conclusion

The availability of mesenchymal stem cells with the potential for the long-term stable expression of a genetic marker has important applications for the advancement of stem cell research. MSCs with a fluorescent marker can be used in experiments utilizing transplantation procedures since they can be easily tracked and identified. This study highlights the importance of thorough *in vitro *characterization of genetically marked cell populations before *in vivo *transplantation. In summary, mASCs and mBMSCs from non-transgenic and eGFP transgenic C57Bl/6 mice have very similar characterization profiles. The presence of eGFP in the transgenic cells does not seem to change stem cell properties, thus supporting the notion that these cells can be utilized in future experiments related to stem cell therapy. These results may be interesting to investigators desiring to employ eGFP transgenic murine stem cell lines in experiments related to stem cell therapeutics or regenerative medicine.

## Methods

### Isolation of stem cells

Murine adipose derived stem cells and bone marrow derived stem cells were obtained from C57Bl/6 mice (Jackson Laboratories, Bar Harbor, ME). They were also obtained from the inbred transgenic strain C57Bl/6-Tg(UBC-GFP)30Scha/J that ubiquitously expresses enhanced green fluorescent protein (Jackson Laboratories). Cells were obtained from 3–4 donors of both sexes from each strain. All donors were 2–4 months old and were individually euthanized by CO_2_. All procedures performed conform to the requirements of the Animal Welfare Act and protocols approved by the Institutional Animal Care and Use Committee at Tulane University. All mice were maintained under standard housing conditions in a pathogen-free environment with free access to food and water.

mBMSCs and GFPTgBMSCs were obtained from two femurs and two tibiae from each donor which were removed and cleaned of connective tissue. The ends of each tibia and femur were clipped off to expose the marrow. A syringe was inserted into the bone and complete expansion media (CEM) was pushed through the bone to collect the marrow. The marrow was re-suspended in CEM using a pipet and then filtered through a 70 μm nylon mesh filter to remove any particulates. The mixture was then centrifuged at 400 × g for 10 minutes at 4°C, and the pellet was re-suspended in 3 ml CEM. The cells were then plated in an 8.8 cm^2 ^culture plate and washed with media twice over a period of 6 hours to remove hematopoietic cells. CEM consists of Iscove's Modified Dulbecco's Medium (IMDM, Invitrogen, Carlsbad, CA) supplemented with 9% fetal bovine serum (FBS; Atlanta Biologicals, Atlanta, GA), 9% horse serum (HS; Hyclone Laboratories, Logan UT), 100 U/ml penicillin (Invitrogen), 100 μg/ml streptomycin (Invitrogen), 0.25 μg/ml amphotericin B (Invitrogen), and 12 μM L-glutamine (Invitrogen).

Adherent cells were washed after 24 hours, and fresh CEM was added every 3 to 4 days until cells reached approximately 80% confluency. The cells were then washed with PBS and lifted by incubation with 0.5 ml 0.25% trypsin/1 mM ethylenediaminetetraacetic acid (EDTA; Invitrogen) for 2–5 minutes at 37°C. The trypsin was neutralized in 5 ml CEM, and all the cells (passage 1) were replated into a 56.7-cm^2 ^culture dish. The cells were either frozen in liquid nitrogen or expanded further. For freezing, the cells were re-suspended and frozen in 5% dimethylsulfoxide (DMSO), 15% FBS, and15% HS in IMDM by placement in a -20°C freezer for 1 hour, then a -80°C freezer for 1 hour, and finally stored in liquid nitrogen.

mASCs and GFPTgASCs were isolated from inguinal fat pads. The tissue was washed extensively with PBS containing 200 U/ml penicillin (Invitrogen), 200 μg/ml streptomycin (Invitrogen), and 0.50 μg/ml amphotericin B (Invitrogen). The fat was minced with sterile scalpels in 0.075% Collagenase Type I and incubated with the collagenase for 1 hour. The collagenase was then neutralized with CEM and the mixture was re-suspended with vigorous pipetting. The tissue was then washed with PBS and filtered through a 70 μm nylon mesh filter. The suspension was centrifuged at 400 × g for 4 minutes at 4°C, and the pellet was re-suspended in 2 ml CEM. The cells were then plated on a 20.8 cm^2 ^culture dish, and the media was replaced after 24 hours. Adherent mASCs were cultured in the same manner as mBMSCs.

### mMSC Differentiation

Cells were plated in 6-well plates at 1000 cells/well and incubated in CEM for 3 days. For osteogenesis, the cultures were then incubated in CEM supplemented with 20 mM glycerol phosphate, 50 ng/mL thyroxine, 1 nM dexamethasone, and 50 μM ascorbate 2-phosphate (all from Sigma, St Louis, MO). The media was changed 2 times per week for 2 weeks. The cells were fixed with 10% formalin for 20 minutes at RT and stained with Alizarin Red, pH 4.1 (Sigma) for 20 minutes at RT.

For the quantitative osteogenesis assay, cells were plated in 96-well plates at 100 cells/well and cultured as aforementioned for 2 weeks. The cells were then stained with Alizarin Red and de-stained with 10% cetylpyridinium chloride for 30 minutes. The amount of Alizarin Red was determined by measuring the optical density (OD) of the solution at 560 nm. The results were then normalized to the protein contents of the samples.

For adipogenesis, the cultures were incubated in CEM supplemented with 5 μg/mL insulin, 50 μM indomethacin, 1 μM dexamethasone, and 0.5 μM 3-isobutyl-1-methylxanthine (IBMX; all from Sigma). The medium was changed 2 times per week for 2 weeks. The cells were fixed with 10% formalin for 20 minutes at RT and stained with 0.5% Oil Red O (Sigma) in methanol (Sigma) for 20 minutes at RT.

For the quantitative adipogenesis assay, cells were plated in 96-well plates at 100 cells/well and cultured as aforementioned for 2 weeks. The cells were then stained with Oil Red O and de-stained with isopropyl alchohol. The amount of Oil Red O was determined by measuring the OD of the solution at 520 nm. The results were then normalized to the protein contents of the samples.

For chondrogenesis, a pellet culture system was used as previously described [24]. Approximately 2 × 10^5 ^mMSCs (passage 8 or lower) were placed in a 15-mL polypropylene tube (Falcon, Bedford, MA), and pelleted by centrifugation. The pellet was cultured at 37°C with 5% CO_2 _in 500 μL chondrogenic media that contained 500 ng/mL bone morphogenic protein-6 (BMP-6; R&D Systems, Minneapolis, MN) in addition to high-glucose DMEM supplemented with 10 ng/mL transforming growth factor-3 (TGF-β3; R&D Systems), 10^-7 ^M dexamethasone (Sigma), 50 μg/mL ascorbate-2-phosphate (Sigma), 40 μg/mL proline (Sigma), 100 μg/mL pyruvate (Sigma), and 50 mg/mL ITS+ Premix (Becton Dickinson, Bedford, MA; 6.25 μg/mL insulin, 6.25 μg/mL transferrin, 6.25 ng/mL selenious acid, 1.25 mg/mL bovine serum albumin, and 5.35 mg/mL linoleic acid). The medium was replaced every 3 to 4 days for 21 days. For microscopy, the pellets were embedded in paraffin, cut into 5 μm thick sections and stained with Toluidine Blue Sodium Borate (Sigma).

### FACS analysis

Cells (passage 9 or lower) were trypsinized, collected, washed with PBS, and incubated for 1 hour at 4°C with phycoerythrin (PE)-conjugated antibodies against Sca-1 (Beckton Dickinson [BD]), CD34 (eBiosciences), CD106 (vascular adhesion molecule-1 [VCAM-1]) (Abcam), CD11b (BD), CD45 (BD), IgG2α (BD), IgG2β (BD), or IgG1 (Chemicon). Excess antibody was removed by washing cells with PBS, and cells were fixed in 1% para-formaldehyde. Detection of PE labeling was performed on a FACScalibur cytometer (BD, San Jose, USA), and results were analyzed using FlowJo software.

### Examination of Growth Rates

Cells (passage 9 or lower) were plated at 100 cells/cm^2 ^in a 56.7 cm^2 ^culture dish in triplicate in CEM and cultured for 12 days. The media was changed every 3 days, and cells were trypsinized and pelleted every 3 days to analyze fold increase in density with a hemacytometer.

### Colony Forming Unit Assay

In order to perform a colony forming unit (CFU) assay, cells (passage 9 or lower) were plated at a density of 100 cells in a 56.7 cm^2 ^culture dish (1.76 cells/cm^2^). The cells were cultured for 14 days with fresh media added after 7 days. The plates were then washed with PBS and stained with 3% Crystal Violet at room temperature for 30 minutes. All colonies greater than 2 mm in diameter were counted. The CFU assay was performed in triplicate for each donor from three different frozen vials of cells.

## Authors' contributions

CR performed all experiments under the supervision of BB. Both authors prepared, edited, and approved the final manuscript.
